# Preoperative Embolization of a Giant Solitary Fibrous Tumor of the Pleura: A Case Report

**DOI:** 10.7759/cureus.30173

**Published:** 2022-10-11

**Authors:** Johan Sebastian Lopera Valle, David Jiménez Marín, José Miguel Hidalgo Oviedo

**Affiliations:** 1 Interventional Radiology, San Vicente Fundación, Medellín, COL; 2 Medicina General, Unidad Médica Integral (UMI), Marinilla, COL; 3 Interventional Radiology, Pablo Tobón Uribe Hospital, Medellín, COL

**Keywords:** case report, thoracotomy, therapeutic, embolization, pleural, solitary fibrous tumor

## Abstract

Only 5% of pleural neoplasms are fibrous tumors of the pleura, which typically develop from sub-mesothelial mesenchymal tissue of the visceral pleura. These tumors often behave clinically benignly, and when they are larger than 15 cm or occupy more than 40% of the hemithorax, they are referred to as “giant” tumors. Surgical excision is the gold standard treatment, although intra-operative bleeding is one of the major complications.

In this case report, we discuss a 39-year-old female patient with a large homogeneous enhancing mass of soft tissue density in the right lower hemithorax with systemic arterial supply from the right inferior phrenic artery. Angiography and embolization were valuable adjuncts in preoperative management. Via thoracotomy, the mass was successfully removed completely with minimal blood loss.

Giant SFTP is a rare neoplasm of the pleura. Intraoperative bleeding is one of the main complications during surgical resection, which is the gold standard of its treatment. Angiography and embolization are valuable complements in the preoperative treatment of this type of tumors to reduce intraoperative blood loss and operative times.

## Introduction

Only 5% of pleural neoplasms are solitary fibrous tumors of the pleura (SFTP), an uncommon neoplasm that develops from sub-mesothelial mesenchymal tissue of the pleura [1,2]. Diagnoses of these tumor forms are sometimes unintentional because of their slow growth. The gold standard of treatment is surgical excision; however, one of the main risks is intraoperative hemorrhage. Prior to surgery, the tumor's vascularity must be evaluated to maximize surgical care and minimize intraoperative and postoperative problems [3].

## Case presentation

**Figure 1:**
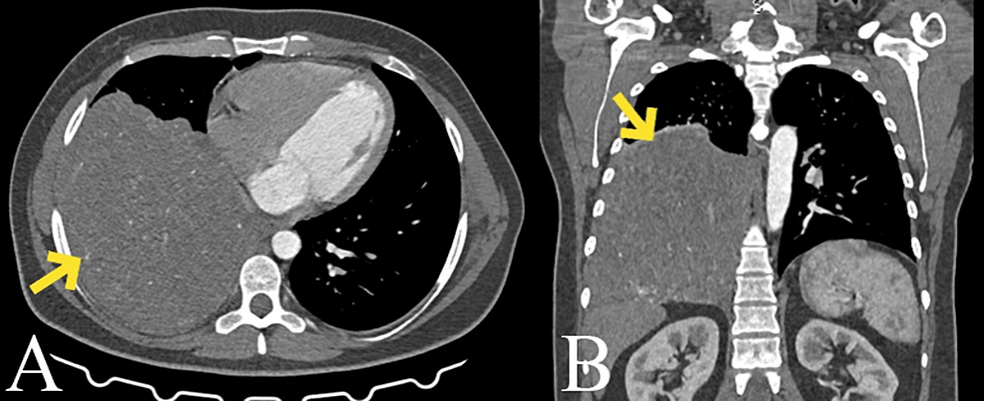
Arterial phase IV contrast enhanced axial acquisition 64 detector CT of the thorax showing a very large homogeneous mass in the right hemithorax. Non-enhancing areas corresponding to necrosis, myxoid degeneration, or hemorrhage within the tumor are not present. (A) Axial. (B) Coronal.

A 39-year-old female presented to the emergency services with complaints of the right lower side of the chest. Her medical history was non-contributory. Upon inspection, it was discovered that her right infra-axillary and infrascapular regions lacked breath sounds. After looking into it, an x-ray of the chest showed signs of a loculated pleural effusion on the right side. A massive homogeneous enhancing mass of soft tissue density of around 14.7 x 15.4 x 13.5 cm was seen in the right lower hemithorax on a contrast-enhanced computed tomogram of the chest. There was no suggestion of a major concomitant mediastinal shift, invasion of the chest wall, or mediastinal lobulation, and the mass was smooth, well-defined, and lobulated. There was no mediastinal adenopathy or pleural effusion that was associated. Additionally, neither necrosis nor calcification was visible (Figures 1A, 1B).

**Figure 2:**
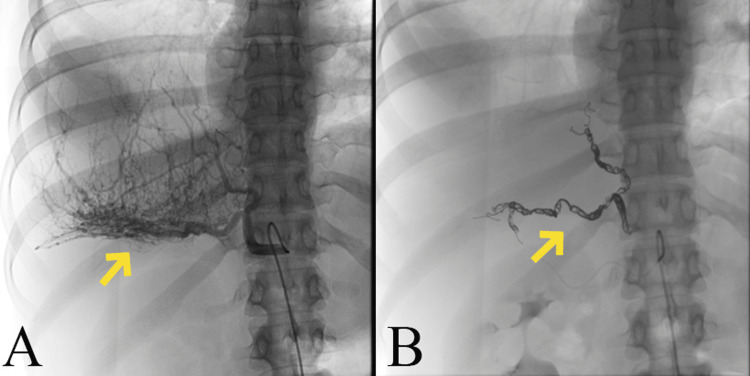
Preoperative embolization of the hypervascular chest tumor. (A) Angiogram shows systemic arterial tumor supply from the right inferior phrenic artery. (B) Imaging following embolization with 300-500 μm polyvinyl alcohol particles and coils showing no residual tumor vascularity.

Preoperative transarterial embolization was performed to facilitate surgical removal of the tumor and to reduce intraoperative blood loss and operative times. Selective angiography with a Simmons 2 diagnostic catheter was performed after common femoral artery access. The right inferior phrenic artery, the unique systemic arterial tumor supply, was embolized by detachable embolization coils (2-3 mm diameters) and 300-500 μm nonspherical polyvinyl alcohol particles (three vials) through a microcatheter 2.7 Fr. Subsequently, the mass was successfully resected via thoracotomy two days later with minimal blood loss (100 mL) (Figures 2A, 2B).

**Figure 3:**
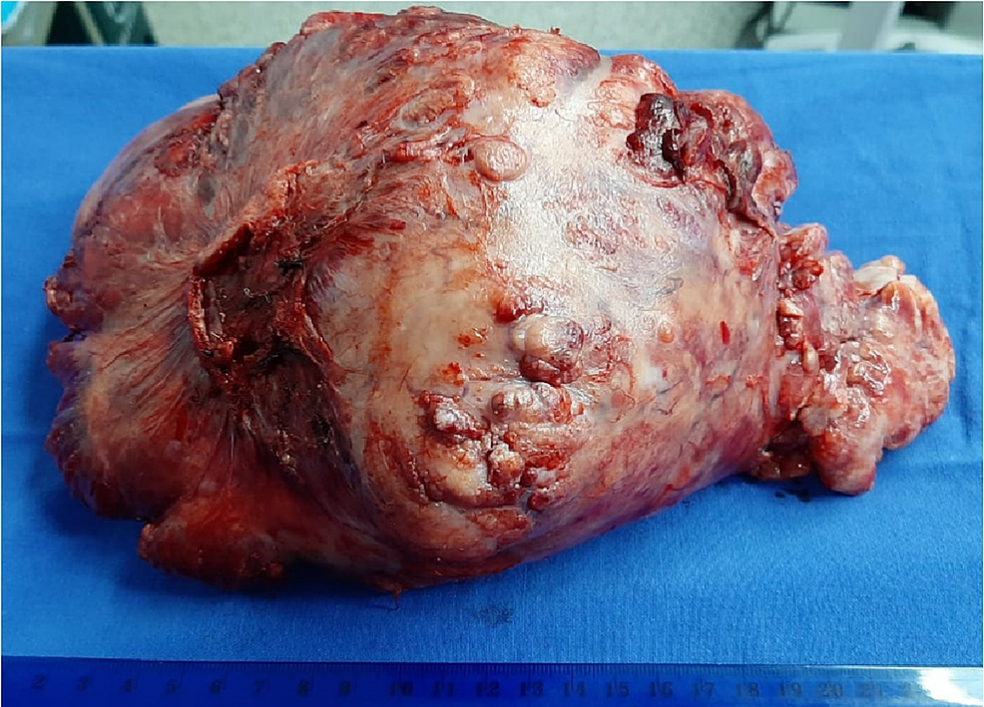
Macroscopic examination showed a large, smooth-surfaced, and nodular tumor measuring 22x13x13 cm, weight 1,587 g.

**Figure 4:**
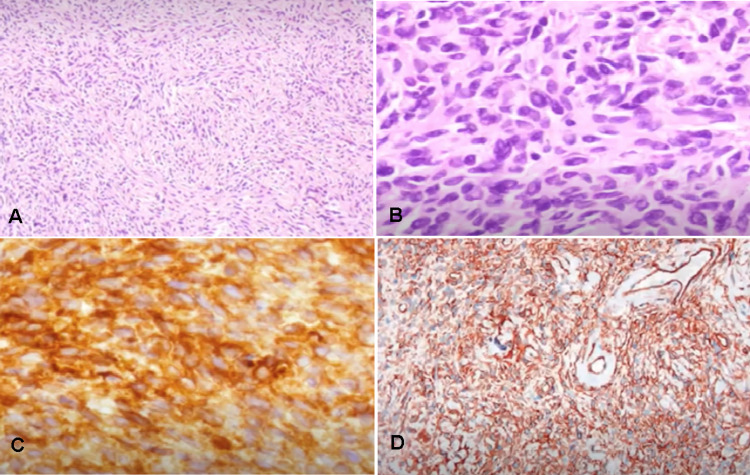
Patternless spindle tumor cells mixed with varying amounts of collagen and hyalination of fibrous tissue that were evidenced in light microscopy (A) H and E × 100. (B) H and E × 400. Positive immunoreactivity of tumor cells, CD34 (C) and Vimentin (D).

Macroscopic examination of the resected tumor showed a large, smooth-surfaced, nodular tumor, which on the cut section was solid with areas of necrosis (Figure 3). Light microscopy revealed an encapsulated spindle cell neoplasm with hemangiopericytic, patternless, and hyaline areas. The less cellular areas contained spindle cells enmeshed in collagen bundles and the cellular areas comprised cells with round to ovoid nuclei with a fine chromatin pattern and pale eosinophilic cytoplasm. No features of malignancy were seen despite extensive sampling and the mitotic rate was less than one per 10 high-power fields. The immunohistochemical profile showed diffuse positivity for CD34 and vimentin (Figures 4A-4D).

According to the morphology and cellular immunophenotype, the diagnosis of benign giant SFTP was signed out. The patient made an uneventful postoperative recovery and was discharged home on the sixth postoperative day.

## Discussion

SFTP is a mesenchymal tumor that tends to affect the pleura, which was initially described by Wagner in 1870 and characterized in 1931 by Kempler [4,5]. The SFTP is a mesenchymal tumor that typically affects the pleura, though it has also been reported in various thoracic (mediastinum, pericardium, and lung) and extrathoracic locations (meninx, epiglottis, salivary glands, thyroid, kidneys, and breast) [6]. With no sex preference, the highest occurrence occurs between the fifth and eighth decades [6]. Only 5% of pleural neoplasms are SFTP [1,2]. About 87 % of cases exhibit benign clinical behavior [5]. However, about 12% of SFTPs are malignant and finally result in mortality from local recurrence or metastatic illness [7]. Giant fibrous pleural tumors are those that are larger than 15 cm in diameter or that take up more than 40% of the hemithorax [8].

At the time of diagnosis, most patients are usually asymptomatic and are usually detected as an incidental finding on a routine chest x-ray. Patients who do have symptoms, it is due to the mass effect on adjacent structures, but these tend to be nonspecific chest symptoms such as chest pain, dyspnea, cough, and, rarely, hemoptysis [6,9]. A cutting-needle biopsy allows for a precise preoperative diagnosis, although most cases are diagnosed by postoperative histology and immunohistochemical examination of the dissected sample [6].

A well-circumscribed lobular homogeneous mass with obvious heterogeneity and necrosis, as well as atelectasis and displacement of other structures, is frequently how an SFTP appears on a CT scan. Although no specific computed tomographic characteristics for the diagnosis of SFTP have been documented, the tumors often show up in contact with the pleural surface [9]. On T1-weighted images, fibrous tissue, which is present in both benign and malignant SFTP, has a low signal intensity. On T2-weighted images, mature fibrous tissue has a low signal intensity, with few cells and an abundant collagen stroma, whereas malignant fibrosis invariably shows a high signal intensity due to increased vascularity, edema, and cellularity. Unfortunately, intratumoral necrosis or myxoid degeneration frequently results in areas of high signal intensity on T2-weighted imaging, rendering benign SFTP difficult to distinguish from malignant SFTP [7]. Pleural effusions only happen in less than 10% of patients, making them quite uncommon [9].

In 38%-50% of cases, the tumor is joined to the pleura by a highly vascular pedicle [3]. The submesothelial connective tissue is where they develop. They may develop from the visceral pleura as well as the parietal pleura, with two-thirds of them coming from the visceral pleura and the final third from the parietal pleura. Eighty percent of tumors start in the visceral pleura but do not extend there. Sessile and pedunculated tumors are split evenly in half [1,10]. Collateral branches from the phrenic artery, the intercostal arteries, internal mammary arteries, and bronchial arteries are frequently responsible for ensuring the tumor's blood supply [3].

Complete surgical excision is the mainstay of therapy and is curative in almost all benign lesions. The considerable bronchial and systemic vascular supply frequently makes surgical excision difficult. Transcatheter arterial embolization may be useful in circumstances where the tumor has a systemic arterial supply to reduce the blood supply and the risk of perioperative bleeding [1].

Authors' search of Pubmed and Google Scholar revealed 82 cases of giant SFTP reported between 1980 and 2014 [3]. Surgical excision was performed on every case described in these studies. In these cases, angiography with embolization was the preferred method; to restrict vascular supply, either alone or in combination, micro-coils, polyvinyl alcohol particles, gelatin sponge, or intravascular plugs were utilized [3]. Ischemic myelitis, which can cause paralysis, other non-target organ ischemia, or thrombotic events after catheter insertion is the main risk of embolization. These are less likely than the risk of exsanguination from the tumor if the careful angiographic technique is used, taking great care to prevent the reflux of embolic agents into the arterial distribution [2]. The average intraoperative bleeding was between 800 and 1,908.3 mL, the amount of bleeding was related to the time of surgery after embolization. Other perioperative complications observed were pleural effusion, air leaks, and redilation pulmonary edema [3]. In the preoperative care of big chest tumors, particularly those with a propensity to a pedicled attachment having extensive vascular systems, like SFTP, transcatheter arterial embolization is a useful adjunct [1].

## Conclusions

Giant SFTP is a rare neoplasm of the pleura, around 50% of cases have a highly vascularized pedicle from which the tumor adheres to the pleura, which makes intraoperative bleeding one of the main complications during surgical resection, the gold standard of its therapy. As seen in our case, preoperative treatment of this type of tumor with transcatheter arterial embolization helps to minimize intraoperative blood loss and surgical time.
